# Renovascular Hypertension with Superimposed Aortic Arch Baroreceptor Failure: Case Report and Review of Literature

**DOI:** 10.1155/2022/4754027

**Published:** 2022-01-21

**Authors:** Amro Daoud, Bisher Mustafa, Hamza Alsaid, Zeid Khitan

**Affiliations:** ^1^Joan C. Edwards School of Medicine-Marshall University, Internal Medicine Department, USA; ^2^Hadassah University Hospital, Al-Quds School of Medicine, Department of Internal Medicine, P.O. Box 17233, Jerusalem, Israel; ^3^Joan C. Edwards School of Medicine-Marshall University, Nephrology Department, USA

## Abstract

**Background:**

Atherosclerotic renal artery diseases are among the most common causes of secondary hypertension. Baroreceptors, as carotid and aortic, are important regulatory mechanisms of blood pressure; their disruption can lead to labile blood pressure due to sympathetic overactivity: an entity called neurogenic hypertension. A disease such as aortic dissection can lead to a challenging combined etiology of secondary hypertension. It can affect both or one of the renal arteries leading to a renovascular pathology that can cause hypertension through RAAS activation. Also, surgical repair of the dissected aortic arch can disrupt baroreceptors leading to neurogenic hypertension. *Case Report*. We report a case of an 83-year-old female patient investigated for recurrent episodes of aphasia. She has a history of hypertension and coronary artery disease. Surgical history is significant for aortic valve replacement complicated by type A aortic dissection requiring surgical repair. Following surgery, the patient developed difficult-to-control and labile blood pressure. Workup included a CT angiogram of the abdominal aorta that showed an infrarenal dominant abdominal aortic aneurysm with juxtarenal aortic dissection; these findings were similar to previous findings. A diagnosis of aortic baroreceptor failure following aortic dissection repair was established, which lead to labile hypertension with superimposed renovascular pathology due to unilateral compromised renal artery blood flow following aortic dissection and thrombosis.

**Conclusions:**

This report highlights the importance of accurate diagnosis of secondary hypertension and its underlying mechanisms, as this has a huge impact on the choice of therapy to avoid undertreatment or overtreatment of hypertension.

## 1. Introduction

Five to ten percent of patients labeled with primary hypertension may have an underlying and possible reversible cause [1]. These causes vary by age. Atherosclerotic renal artery diseases are considered among the most common causes that can lead to secondary hypertension in adults aged 65 years or older [1].

Aortic dissection can cause reduced blood flow to one or both kidneys, in a mechanism close to atherosclerotic renal artery disease, which can lead to severe and uncontrollable hypertension [2]. The reduced blood flow activates the renin-angiotensin-aldosterone system (RAAS) and hence the elevation in blood pressure [3]. Another entity of secondary hypertension is neurogenic hypertension, which is driven by increased activity of the sympathetic nervous system that plays a major role in such patients due to affecting both neurological and renal factors that contribute to elevated blood pressure [4]. In these patients, stimulation of beta-1 receptors in the heart and renal tissue will lead to increased heart rate and increased renin secretion by juxtaglomerular cells, respectively [4]. Also, stimulation of alpha receptors in the arteries will lead to vasoconstriction and increased peripheral vascular resistance, in addition to reduction in glomerular filtration rate and renal blood flow which in turn will also increase renin secretion [4].

Baroreceptors in the adventitia of the carotid body and aortic arch, which consist of mechano-sensitive terminals of the vagus and glossopharyngeal nerves that terminate into the nucleus tractus solitarius (NTS) in the caudal medulla, are part of the regulatory mechanism of blood pressure by sensing the grade of distension of the vessels [5, 6]. They play an important role in buffering blood pressure through alternating the activation and inhibition of parasympathetic and sympathetic nervous system [7].

When this regulatory mechanism is disrupted, a resulting labile blood pressure is expected, which can range from hypercatecholaminergic tone causing severe hypertension to the contrasting episodes of profound hypotension [5]. Here, we are presenting a case that has two potential causes of secondary hypertension discussing pathophysiology and treatment.

## 2. Case Presentation

An 83-year-old female with a history of hypertension, coronary artery disease, and dementia was investigated for recurrent brief episodes of aphasia.

Her past surgical history is significant for bioprosthetic aortic valve replacement 8 years prior to this presentation which was complicated by type A aortic dissection (ascending aortic dissection propagated to the abdominal aorta and right common and external iliac arteries) for which she underwent surgical repair of the thoracic aorta. Around two months following the surgery, the patient was noted to have labile and poorly controlled blood pressure and was treated with amlodipine and losartan.

Physical examination revealed blood pressure of 176/84 mmHg and a heart rate of 70 bpm. There was no focal neurological deficit, heart murmurs, vascular bruits, palpable masses, or blood pressure discrepancy between the upper and lower extremities.

Laboratory studies including complete metabolic panel and complete blood count were nonsignificant.

**Figure 1:**
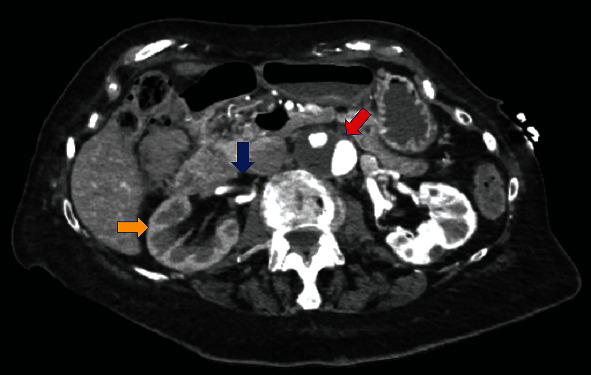
CTA of the aorta of the patient with bilateral run off showing infrarenal dominant abdominal aortic aneurysm with juxtarenal aortic dissection (red arrow); it also shows that the false lumen supplies the right renal artery which is mostly thrombosed (blue arrow), in addition to delayed right kidney nephrogram (yellow arrow).

During hospital stay, acute stroke was ruled out and her presenting symptoms were attributed to hypertensive encephalopathy. Among stroke workup, the patient had an echocardiogram that was concerning for descending aorta questionable thrombus. CT angiogram (CTA) of the abdominal aorta with bilateral femoral run off was done (as shown in Figure 1) and showed infrarenal dominant abdominal aortic aneurysm with juxtarenal aortic dissection; it also showed that the false lumen supplies the right renal artery which was mostly thrombosed.

Old medical records were obtained and showed similar findings on previous imaging, and the impression was that this dissection is the same as the old one, with thrombosis in the false lumen, and no further management or treatment specific to the dissection was indicated.

**Figure 2:**
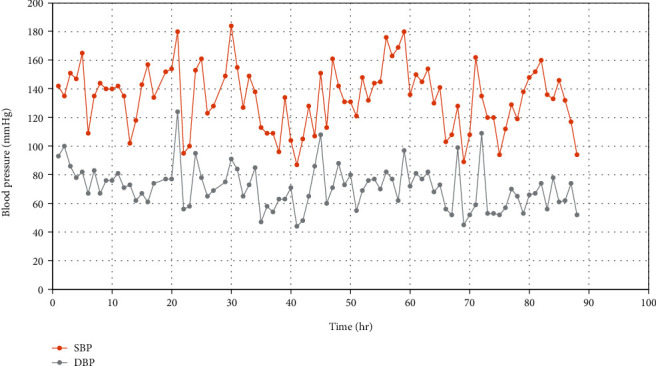
Blood pressure values monitored over a period of approximately 90 hours during hospitalization showing variations in an hour-to-hour readings of blood pressure. hr: hour; SBP: systolic blood pressure; DBP: diastolic blood pressure.

Patient's blood pressure was noticed to be labile during hospitalization (as shown in Figure 2), and in the light of imaging findings, a renovascular hypertension due to right kidney decreased blood flow with superimposed defective aortic baroreceptor mechanism due to aortic arc surgery was suspected to be causing her poorly controlled labile blood pressure.

As a result, the patient was started on oral labetalol in addition to her home losartan. We also discontinued her home amlodipine. After that, her blood pressure was controlled without hypotensive episodes, and the patient was discharged home.

## 3. Discussion

Maintenance of arterial blood pressure requires intact autoregulatory feedback system with functioning sensing and effector mechanisms. Baroreceptors are mechanoreceptors that sense physical distortion of the blood vessels as in high-pressure carotid, aortic arch and afferent arteriole baroreceptors and low-pressure baroreceptors located within the atria, ventricles, and pulmonary vasculature [6, 8]. On the other hand, chemoreceptors located in the macula densa of the juxtaglomerular apparatus are considered the sensory arm of the renin-angiotensin-aldosterone system (RAAS) that senses delivery of sodium chloride and determines downstream function [9]. Moreover, another set of chemoreceptors located in the carotid body is able to sense changes in arterial partial pressures oxygen and carbon dioxide (PO_2_, PCO_2_) and pH have been shown to affect blood pressure regulation in health and disease [10].

**Figure 3:**
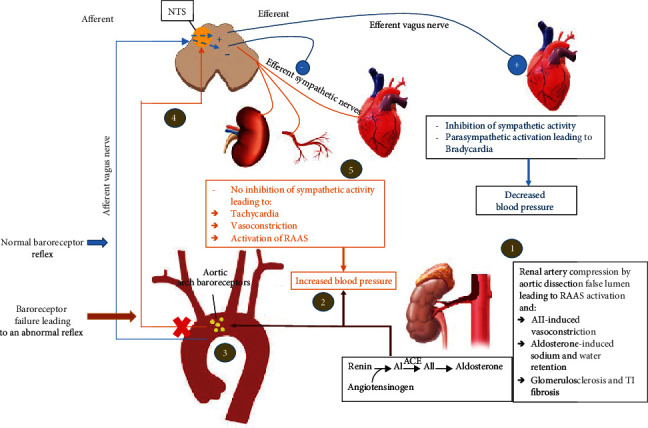
Schema representing the exaggeration in arterial BP in the setting of RVH and aortic arch baroreceptor failure. The rise in BP resulting from RVH (steps 1 and 2) is not transmitted to the NTS from aortic baroreceptors (steps 3 and 4). This results in the maintenance of efferent sympathetic outflow to the heart, kidneys, and blood vessels leading to further rise in blood pressure (step 5). Blue color: body response to elevated blood pressure via normal aortic arch receptors. Orange color: body response to hypertension in the setting of interrupted aortic arch receptors. BP: blood pressure; RVH: renovascular hypertension; NTS: nucleus tractus solitarius; AI: angiotensin I; AII: angiotensin II; ACE: angiotensin converting enzyme; RAAS: renin-angiotensin-aldosterone system.

While neurological excessive tone can be a component of primary hypertension [11], efferent outputs are generated in response to the sensed alterations in blood pressure through the RAAS and the sympathetic nervous system to help restore blood pressure [12]. These outputs act by modifying the peripheral vascular resistance and cardiac output. Activation of RAAS in response to hypotension results in increased production of angiotensin II and aldosterone with resulting vasoconstriction and sodium and water retention [12]. Alternatively, an increase in arterial blood pressure will result in an increase in the rate of impulse firing from the baroreceptors to the nucleus tractus solitarius (NTS) resulting in a decrease in the sympathetic outflow to the peripheral arterial system, heart, and the kidneys as shown in Figure 3 [7, 8]. This results in vasodilatation of the blood vessels, decrease in heart rate and myocardial contractility, and decrease in the rate of renin secretion by the juxtaglomerular apparatus. It is the integrity of the afferent and efferent mechanism in autoregulation that maintains the arterial blood pressure within a narrow range.

In renovascular hypertension, the poor blood flow to the kidney results in activation of the RAAS that subsequently leads to elevation in blood pressure [12, 13], and 8% of cases with aortic dissection has associated renal malperfusion [13] as our patient does have to her right kidney.

In this case, it is expected that functioning baroreceptors will exert its negative feedback loop to attenuate blood pressure. In our patient, the interrupted afferent flow from the aortic baroreceptors to the NTS following aortic arch repair following dissection resulted in the exaggeration of hypertension. The onset of poor blood pressure control following aortic arch surgery supports this association. Baroreflex failure has been described in literature before, and mostly was due to neck trauma, extensive neck surgery, or radiation therapy for cancers located in the neck [7]. In our case, we are describing a baroreflex failure due to aortic arch surgery. Baroreflex failure not only can cause episodes of hypotension, it can also lead to episodes of extensively elevated blood pressure that can be accompanied by tachycardia [7]. On the other hand, hypotensive episodes can occur at rest when cortical input is diminished [7], and both of these phenomena are present in our case. In cases of baroreceptor interruption and failure, patients are thought to experience hypotensive episodes with vasodilating drugs such as calcium channel blockers [7], so the decision was made to discontinue home Amlodipine and to start labetalol 100 mg twice daily to counteract inappropriate sympathetic activation by the failed baroreceptors that cause tachycardia in addition to RAAS activation through beta antagonist activity of labetalol, in addition to counteracting the increased systemic vascular resistance through its alpha antagonist activity. Also, we resumed home losartan 100 mg daily to treat the other contributing factor of our patient's hypertension, which is right renal stenosis, as RAAS blockade is one of the proven and preferred medications to treat renovascular hypertension particularly in unilateral renovascular disease [14]. In cases of atherosclerotic renal artery stenosis, several reports showed no significant benefits of revascularization stent placement over medical treatment in patients with atherosclerotic renovascular disease in terms of mortality, kidney function, and blood pressure control [15, 16]. In our report, a revascularization was unsuitable and unnecessary as the patient's blood pressure was controlled by medical treatment alone, and since many years passed with vascular compromise on the right kidney which could have affected its function given the delayed nephrogram on CTA, it is less likely that surgical repair to relieve a thrombosed false lumen supplying the renal artery would have benefitted an already compromised kidney for many years.

## 4. Conclusion

We report a case of aortic baroreceptor failure after aortic dissection repair surgery causing labile hypertension with superimposed renovascular pathology due to unilateral compromised renal artery blood flow secondary to aortic dissection and thrombosis. This case report highlights the importance of accurate diagnosis of secondary hypertension and its underlying mechanisms, as this has a huge impact on the choice of therapy to avoid undertreatment or overtreatment of hypertension.
